# The Impact of the Preferred Reporting Items for Case Reports in Endodontics (PRICE) 2020 Guidelines on the Reporting of Endodontic Case Reports

**DOI:** 10.7759/cureus.37553

**Published:** 2023-04-14

**Authors:** Sofian Youssef, Phillip Tomson, Amir Reza Akbari, Natalie Archer, Fayjel Shah, Jasmeet Heran, Sunmeet Kandhari, Sandeep Pai, Shivakar Mehrotra, Joanna M Batt

**Affiliations:** 1 Academic Research, University of Nottingham, Nottingham, GBR; 2 Endodontics, University of Birmingham, Birmingham, GBR; 3 Obstetrics and Gynecology, King’s Mill Hospital, Mansfield, GBR; 4 Restorative Dentistry, University of Birmingham, Birmingham, GBR

**Keywords:** clinical dentistry, case report series, guideline, clinical case report, endodontic

## Abstract

Aim

The aim of this study is to evaluate the impact of the Preferred Reporting Items for Case reports in Endodontics (PRICE) 2020 guideline on the reporting of published endodontic case reports (CRs).

Methodology

All case reports published in the International Endodontic Journal, European Endodontic Journal, Journal of Endodontics and Restorative Dentistry and Endodontics, in the year before and after the release of PRICE 2020, were included for analysis. Two panels comprising dentists scored case reports against a scoring system adapted from the guideline. Individual items were scored up to a maximum of 1; scores were then summated to provide an overall maximum of 47 for each CR. Each report provided an overall percentage adherence, and panel agreement was calculated using the intraclass correlation coefficient (ICC). Disagreement on scoring was discussed until a consensus was reached. Scores before and after PRICE guideline publication were compared using an unpaired two-tailed t test.

Results

A total 19 CRs were identified in both the pre- and post-PRICE guideline publication. Mean adherence to PRICE 2020 increased by 7.9% (p=0.003) from 70.0%±8.89 to 77.9%±6.23 following its publication. Agreement between panels was moderate (ICC pre-PRICE: 0.673 {p=0.011}; ICC post-PRICE: 0.742 {p=0.003}). Items 1a, 6c, 6e, 6f, 6g, 6j, 6q, 6s, 7a, 9a, 11a, 12c and 12d experienced a fall in compliance.

Conclusion

The PRICE 2020 guideline has resulted in a modest improvement in the reporting of endodontic case reports. Greater awareness and a wider acceptance and implementation of the guideline in endodontic journals are needed to improve adherence to the novel guideline.

## Introduction

Case reports (CR) provide detailed accounts of signs, symptoms, diagnosis and management of rare presentations or novel management techniques in order to share different aspects of clinical practice of interest [1]. The current classification of CRs is level 4 (poorest level) as per the Oxford Centre for Evidence-Based Medicine: Levels of Evidence [2]. Although CRs are graded as one of the lowest levels of evidence, they provide value in the rapid communication of rare incidents, conditions and associations, which may inform further higher-quality research. The lack of consistency, transparency, information and coherency in the reporting of CRs can result in poor educational value and weaker evidence. As a result, case reporting recommendations were introduced in 2013 in the form of CAse REport (CARE) guidelines, providing a 13-item (30 individual points) detailed checklist for authors to follow [3]. In recent years, specialties have adapted CARE guidelines using Delphi consensus methodology to provide tailored frameworks for respective practices [4,5]. Endodontic case reporting practice is currently lacking; unpublished data by Dummer, reported by Nagendrababu et al., elucidates that a significant portion of CRs is rejected due to ‘incomplete’ and ‘inaccurate’ reporting. The Preferred Reporting Items for Case reports in Endodontics (PRICE) 2020 were therefore created with the intention of improving writing practices and enhancing the educational value of CRs [6].

PRICE introduced a comprehensive 47 individual-item checklist divided into 12 sections that are far more detailed than most case reporting guidelines (CARE: 30 individual items [3]; SCARE: 38 individual items [4]). Items were first developed in draft format, by integrating and adapting fundamental points from CARE [3] and the Clinical and Laboratory Images in Publications (CLIP) principles [7]. CLIP was introduced in 2012 in a concerted effort to promote the publication of clearer and more informative images, such as histopathology and radiography. PRICE was introduced to provide guidance for reporting singular case reports with no mention of case series articles. Draft PRICE items were reviewed via an online Delphi survey, where a group, comprising academics, endodontists, general dentists and patient representatives, scored items for inclusion. The findings from the Delphi survey and the revised PRICE items were thereafter discussed at an international endodontic meeting (19th European Society of Endodontology Biennial Congress), where the guideline was finalised. The combined emphasis on the reporting of both cases (CARE) and images (CLIP) makes PRICE the first of its kind, as previous recommendations have placed little emphasis on the reporting standards of images. PRICE also introduces a comprehensive flowchart for authors, summarising the process of writing a thorough and detailed case report [6].

Contemporaneous evaluations of endodontic case reporting practice have exemplified the need for PRICE [8]. Reporting guidelines are particularly necessary for the purposes of promoting clearer, more informative and uniform writing practice. A significant limitation to achieving such outcomes remains the authors’ awareness, understanding and interpretation. The evaluations of both SCARE, guideline for the reporting of surgical case reports, and Preferred Reporting Of CasE Series in Surgery (PROCESS), guideline for the reporting of surgical case series, elucidated improvements in reporting practices [4,5,9]. Given the index of potential guidelines that possess influencing positive change, consistent long-term auditing of reporting practice is warranted. The present study therefore aimed to evaluate the early impact of the PRICE guidelines on the reporting of endodontic case reports in four reputable endodontic scientific journals.

## Materials and methods

Study design

A before-and-after study was conducted, evaluating adherence to endodontic CRs published prior to and following the publication of PRICE guidelines (23 February 2020) [6]. Two panels, comprising three dentists with interests in endodontics, independently scored CRs against the PRICE scoring system (Table 1), based on the detailed exposition and elaboration of items present in PRICE [10]. Each individual item was allocated a maximum score of 1 (e.g. item 6a=maximum of one and item 6c=maximum of one). Two independent panels were used to strengthen the reliability of CR scores. Each panel of three scored all the CRs as a group; a disagreement on finalised item scoring between panels was settled by discussion between both panels until a consensus was reached. Ethical approval was not required for this before-and-after study of published case reports; individual patient consent was obtained by the authors of respective CRs prior to publication.

**Table 1:** Scoring criteria to assess endodontic case report adherence to PRICE 2020 reporting guidelines. The scoring criteria to assess endodontic case report adherence to PRICE 2020 reporting guidelines were formulated using Nagendrababu et al.’s explanation and elaboration of reporting guidelines [10]. PRICE, Preferred Reporting Items for Case reports in Endodontics; MeSH, Medical Subject Heading; NA, not available

Items		PRICE Guideline Consensus Criteria	Review Scoring Criteria
Titles			
Item 1	Item 1a	The word ‘case report(s)’ must be included in the title	1 for ‘case report’ in the title; 0 for not mentioned
	Item 1b	The area of interest (e.g. anatomy, disease and treatment) must be included briefly in the title	1 for the area of interest described in the title; 0 for not mentioned
Keywords			
Item 2	Item 2a	At least two relevant keywords, preferably MeSH terms, related to the content of the case report must be included	1 for two relevant keywords; 0 for not mentioned
Abstract			
Item 3	Item 3a	The introduction must contain information on how the report is novel and contributes to the literature and clinical practice and/or fills a gap(s) in knowledge	1/2 for a statement regarding novelty; 1/2 for a statement regarding contribution to the literature/knowledge/practice; 0 for not mentioned
	Item 3b	The body must describe the main clinical findings, including symptoms and signs, if present	1 for clinical finding description; 0 for not mentioned
	Item 3c	The body must describe the main radiographic/histological/laboratory/diagnostic findings	1 for describing the key finding of the report; 0 for not mentioned
	Item 3d	The body must describe the main outcomes of the treatment, if active treatment has been provided	1 for the description of management; 0 for not mentioned
	Item 3e	The conclusion(s) must contain the main ‘take‐away’ lesson(s), sometimes referred to as key learning point(s)	1 for a statement summarising the key learning point; 0 for not mentioned
Introduction			
Item 4	Item 4a	A background summary of the case(s) with relevant information must be provided	1 for a summary of the case within the introduction; 0 for not mentioned
Informed consent			
Item 5	Item 5a	Informed consent, a clear statement that informed, valid consent was obtained from the patient(s), must be provided	1 for a clear statement regarding informed patient consent; 0 for not mentioned
Case report information			
Item 6	Item 6a	The age of the patient(s) must be provided	1 for age; 0 for not mentioned
	Item 6b	The gender of the patient(s) must be provided	1 for gender; 0 for not mentioned
	Item 6c	The ethnicity of the patient(s) must be provided, if relevant	1 for ethnicity; 0 for not mentioned; NA for not relevant
	Item 6d	The main concern, chief complaint or symptoms of the patient(s), if any, must be provided	1 for main presenting complaint; 0 for not mentioned
	Item 6e	The medical history of the patient(s) must be provided, if relevant	1 for relevant medical history; 0 for not mentioned; NA for not relevant
	Item 6f	The dental history of the patient(s) must be provided, if relevant	1 for relevant dental history; 0 for not mentioned; NA for not relevant
	Item 6g	The family history of the patient if associated with the primary complaint (PC) must be provided, if relevant	1 for relevant family history; 0 for not mentioned; NA for not relevant
	Item 6h	The psychosocial history of the patient if associated with the primary complaint must be provided, if relevant	1 for psychosocial history associated to PC; 0 for not mentioned; NA for not relevant
	Item 6i	Genetic information, including details of relevant comorbidities and past interventions and their outcomes, must be provided when possible, if relevant	1 for genetic information (relevant comorbidities and past interventions and their outcomes); 0 for not mentioned; NA for not relevant
	Item 6j	Extra‐oral findings must be provided, if relevant	1 for extra-oral findings; 0 for not mentioned; NA for not relevant
	Item 6k	General intra‐oral findings must be provided when relevant (e.g. carious lesions, restorations, periodontal condition and soft tissues)	1 for intra-oral findings; 0 for not mentioned; NA for not relevant
	Item 6l	Important/relevant dates and times (in the text or a table or figure) must be provided in chronological order	1 for dates and times in chronological order (in text or a table or figure); 0 for not mentioned
	Item 6m	The diagnostic methods and the results for the specific tooth/teeth (e.g. pulp sensibility test, tenderness, mobility, periodontal probing depths, laboratory investigations, imaging techniques or other special tests) must be provided	1/2 for the diagnostic methods for the case; 1/2 for the result of the method; 0 for not mentioned
	Item 6n	The diagnostic challenges, if any, must be provided	1 for the diagnostic challenge explicitly mentioned; 0 for not mentioned; NA for not relevant
	Item 6o	The diagnostic reasoning including other possible diagnoses that were considered must be provided	1 for diagnostic reasoning (including differentials); 0 for not mentioned
	Item 6p	The active treatment(s) or intervention(s) performed, if any, must be provided	1 for the treatment or intervention used; 0 for not mentioned; NA for not relevant
	Item 6q	Any modifications to the proposed treatment(s) or intervention(s), if necessary, must be provided	1 for the modifications of proposed intervention/treatment; NA for not relevant
	Item 6r	The assessment method(s) used to determine the clinician‐assessed and patient‐assessed treatment outcomes and their results must be provided	1/2 for the assessment methods to determine clinician-assessed outcome and result; 1/2 for the assessment methods to determine patient-assessed outcome and result; 0 for not mentioned
	Item 6s	Adverse and unanticipated events or consequences, if any, must be provided	1 for adverse/unanticipated consequences; NA for not mentioned
Discussion			
Item 7	Item 7a	The specific treatment(s) and intervention(s) (if any) must be discussed with reference to the relevant literature	1 for the specific treatment/intervention discussed with reference to the literature; 0 for not mentioned
	Item 7b	The strengths of the case report and its importance must be discussed with reference to the relevant literature	1/2 for the strengths of the report discussed with reference to the literature; 1/2 for the importance of the report discussed with reference to the literature; 0 for not mentioned
	Item 7c	The limitations of the case report must be discussed	1 for the limitations of the report discussed; 0 for not mentioned
	Item 7d	The rationale for the conclusion(s) must be discussed	1 for the rationale of the conclusion discussed; 0 for not mentioned
Patient perspective			
Item 8	Item 8a	Feedback from the patient on the treatment and the care they received should be provided, if relevant	1 for patient feedback on treatment and care; 0 for not mentioned; NA for not relevant
Conclusion			
Item 9	Item 9a	Explicit conclusion(s) (i.e. the main ‘take‐away’ lessons) must be provided	1 for explicit conclusion; 0 for not mentioned
	Item 9b	Implications for clinical practice or future research must be provided	1 for implication on practice/future research; 0 for not mentioned
Funding details			
Item 10	Item 10a	Sources of funding and other support (such as the supply of instruments and equipment), as well as the role of funders, must be acknowledged and described	1 for a statement regarding funding/support; 0 for not mentioned
Conflict of interest			
Item 11	Item 11a	An explicit statement on conflicts of interest must be provided	1 for explicit statement regarding conflicts of interest; 0 for not mentioned
Quality of images			
Item 12	Item 12a	The details of the equipment, software and settings used to acquire the image(s) must be described in the text or legend	1 for the equipment/software/settings used to acquire the image; 0 for not mentioned
	Item 12b	The reason why the image was acquired and the rationale for its inclusion in the manuscript must be provided in the text	1 for the explicit mention of the reasoning behind the inclusion of the image in the manuscript; 0 for not mentioned
	Item 12c	The circumstances (conditions) under which the images were viewed and evaluated by the authors must be provided in the text	1 for the mention of conditions under which the images were viewed and evaluated by the authors; 0 for not mentioned
	Item 12d	The resolution and any magnification of the image(s) or any modifications/enhancements (e.g. adjustments for brightness, colour balance, magnification, image smoothing and staining) that were carried out must be described in the text or legend	1 for a statement regarding resolution and modifications to the image; 0 for not mentioned
	Item 12e	Patient identifiers (names and patient numbers) must be removed to ensure they are anonymised	1 for patient identifiers anonymised; 0 for patient identifiers present in images
	Item 12f	An interpretation of the findings (meaning and implications) from the image(s) must be provided in the text	1 for the interpretation of findings from images provided in text; 0 for not mentioned
	Item 12g	The legend associated with each image must describe clearly what the subject is and what specific feature(s) it illustrates. Legends associated with the images of patients must describe the age, gender and ethnicity of the person, if relevant	1/2 for the legend describing image and features; 1/2 for the legend describing age, gender and ethnicity; 0 for not mentioned
	Item 12h	Markers/labels must be used to identify the key information in the image(s) and be defined in the legend or as a footnote	1/2 for markers to identify key information in the image; 1/2 for the marker defined in the legend; 0 for no markers
	Item 12i	The legend of each image must include an explanation whether it is pre‐treatment, intra‐treatment or post‐treatment and, if relevant, how images over time were standardised	1/2 for a statement of whether the image was pre‐treatment, intra‐treatment or post‐treatment; 1/2 for a mention in the legend of how images were standardised over time; 0 for not mentioned

Case report selection

All endodontic CRs published in the nine months following the publication of PRICE, incorporating a two-month delay, were identified [6]. The same number of CRs was then identified in the two years directly prior to the publication of PRICE in retrospective chronological order until the number of CRs was achieved. CRs were retrieved from the issues of four endodontic journals for the ease of identifying only endodontic CRs: the International Endodontic Journal, European Endodontic Journal, Journal of Endodontics and Restorative Dentistry and Endodontics. For the purposes of this study, only endodontic CRs describing one case were included.

Data analysis

Statistical analyses were performed using the Statistical Package for Social Sciences (SPSS) version 27.0 (IBM SPSS Statistics, Armonk, NY). Individual-item scores were collated to form a final overall score out of 47 for each CR; scores were then converted to percentages, by dividing CR score over maximal applicable scoring (items deemed not applicable were subtracted from the denominator) [5]. Intraclass correlation coefficient (ICC) was used to measure the agreement between final percentage panel ratings. Data was analysed for normality using the Shapiro-Wilk test, and parametric variables from before and after periods were compared using an unpaired two-tailed t test. A p-value of <0.05 was considered significant.

## Results

A total of 19 CRs were identified in both the pre- and post-PRICE periods. The mean score for adherence to the PRICE guideline increased by 7.9% (t=-3.141, degrees of freedom {df}=36 and p=0.003) from 70.0% (standard deviation: ±8.89) in the pre-PRICE period to 77.9% (standard deviation: ±6.23) in the post-PRICE period (Table 2). ICC between panels for pre-PRICE was 0.673 (p=0.011) and post-PRICE was 0.742 (p=0.003), indicating moderate agreement of scoring.

**Table 2:** A Comparison of article adherence to the PRICE guideline before and after their publication in 2020. PRICE: Preferred Reporting Items for Case reports in Endodontics

Pre-PRICE Guideline Articles				
Authorship	Year of Publication	Article Title	Journal	Total Score
Ali and Arslan [11]	2019	Guided endodontics: a case report of maxillary lateral incisors with multiple dens invaginatus	Restorative Dentistry and Endodontics	70.4
Arango-Gómez et al. [12]	2019	Pulp revascularization with and without platelet-rich plasma in two anterior teeth with horizontal radicular fractures: a case report	Restorative Dentistry and Endodontics	77.3
Arbel et al. [13]	2019	Autotransplantation after primary bone repair of a recipient site with a large periradicular lesion: a case report	International Endodontic Journal	78.8
Arslan et al. [14]	2019	Histologic evaluation of regenerated tissues in the pulp spaces of teeth with mature roots at the time of the regenerative endodontic procedures	Journal of Endodontics	71.3
Buchgreitz et al. [15]	2019	Guided endodontics modified for treating molars by using an intracoronal guide technique	Journal of Endodontics	78.5
Cho and Jung [16]	2019	Complete healing of a large cystic lesion following root canal treatment with concurrent surgical drainage: a case report with 14-year follow-up	Journal of Endodontics	70.2
Chung et al. [17]	2019	A case report of multiple bilateral dens invaginatus in maxillary anteriors	Restorative Dentistry and Endodontics	54.5
Deeb et al. [18]	2019	Discontinuation of denosumab as a potential cause of generalized external cervical root resorption: a case report	Journal of Endodontics	73.2
Gambarini et al. [19]	2019	Endodontic microsurgery using dynamic navigation system: a case report	Journal of Endodontics	65.8
Kim et al. [20]	2019	A new minimally invasive guided endodontic microsurgery by cone beam computed tomography and 3-dimensional printing technology	Restorative Dentistry and Endodontics	62.2
Kim et al. [21]	2019	Surgical management of an accessory canal in a maxillary premolar: a case report	Restorative Dentistry and Endodontics	53.7
Krug et al. [22]	2019	Intentional replantation with an atraumatic extraction system in teeth with extensive cervical resorption	Journal of Endodontics	79.7
Llavayol et al. [23]	2019	Multiple cervical root resorption in a young adult female previously treated with chemotherapy: a case report	Journal of Endodontics	73.1
Petitjean et al. [24]	2018	Multimodular assessment of a calcified extraradicular deposit on the root surfaces of a mandibular molar	International Endodontic Journal	59
Ong [25]	2019	Non-surgical retreatment after failed intentional replantation: a case report	European Endodontic Journal	80.2
Shemesh et al. [26]	2019	Cone-beam computed tomography as a noninvasive assistance tool for oral cutaneous sinus tract diagnosis: a case series	Journal of Endodontics	63
de Albuquerque et al. [27]	2019	Treatment of an acute apical abscess in a patient with autoimmune hepatitis taking alendronate: a case report	Journal of Endodontics	83.3
Torres et al. [28]	2019	Microguided endodontics: a case report of a maxillary lateral incisor with pulp canal obliteration and apical periodontitis	International Endodontic Journal	73.6
Yoon et al. [29]	2018	Anatomical analysis of the resected roots of mandibular first molars after failed non-surgical retreatment	Restorative Dentistry and Endodontics	62.5
Post-PRICE Guideline Articles				
Authorship	Year of Publication	Article Title	Journal	Total Score
Arnold [30]	2021	Reparative endodontic treatment of a perforating internal inflammatory root resorption: a case report	Journal of Endodontics	85
Azim et al. [31]	2020	Clinical endodontic management during the COVID-19 pandemic: a literature review and clinical recommendations	International Endodontic Journal	84.1
Bi et al. [32]	2020	Endodontic microsurgery with orthodontic treatment in a mandibular left molar with symptomatic apical periodontitis	Journal of Endodontics	70
Cardoso et al. [33]	2021	Resolution of nasal sinus tract after endodontic therapy: a case report with microbial analysis	Journal of Endodontics	83.7
Cordero et al. [34]	2020	Allogeneic cellular therapy in a mature tooth with apical periodontitis and accidental root perforation: a case report	Journal of Endodontics	75.6
Falcon et al. [35]	2021	Chamberless endodontic access for treatment of calcified anterior central incisors	Journal of Endodontics	77.5
Fráter et al. [36]	2020	Bioblock technique to treat severe internal resorption with subsequent periapical pathology: a case report	Restorative Dentistry and Endodontics	79.1
Kiho et al. [37]	2020	Pulpal disease arising from medication-related osteonecrosis of the jaw: a case report	Journal of Endodontics	80.7
Kim et al. [38]	2020	The application of “bone window technique” using piezoelectric saws and a CAD/CAM-guided surgical stent in endodontic microsurgery on a mandibular molar case	Restorative Dentistry and Endodontics	78.6
Mello et al. [39]	2020	Neuropathy mimicking dental pain in a patient diagnosed with Lyme disease	Journal of Endodontics	71.1
Michaelson et al. [40]	2021	A case report of non-Hodgkin low-grade B-cell mucosa-associated lymphoid tissue lymphoma presenting as a suspected endodontic lesion	Journal of Endodontics	79.8
Mori et al. [41]	2020	Endodontic approach in a replanted tooth with an immature root apex and chronic apical periodontitis: a case report	Restorative Dentistry and Endodontics	61.6
Ricucci et al. [42]	2020	Unusual location of dens invaginatus causing a difficult-to-diagnose pulpal involvement	Journal of Endodontics	79.1
Ricucci et al. [43]	2020	An unusual case of a large periapical cyst mimicking a nasopalatine duct cyst	Journal of Endodontics	74.4
Kim et al. [44]	2020	A novel approach to fracture resistance using horizontal posts after endodontic therapy: a case report and review of literature	Journal of Endodontics	69.8
Schürz et al. [45]	2020	Preservation of a split tooth: nonsurgical clinical management	Journal of Endodontics	85.7
Shilkofski et al. [46]	2020	Non-Hodgkin’s lymphoma of the anterior maxilla mimicking a chronic apical abscess	Journal of Endodontics	83
Strbac et al. [47]	2020	Guided osteotomy and guided autotransplantation for treatment of severely impacted teeth: a proof-of-concept report	Journal of Endodontics	82.1
Torres [48]	2021	Guided endodontics: use of a sleeveless guide system on an upper premolar with pulp canal obliteration and apical periodontitis	Journal of Endodontics	78.6

**Figure 1:**
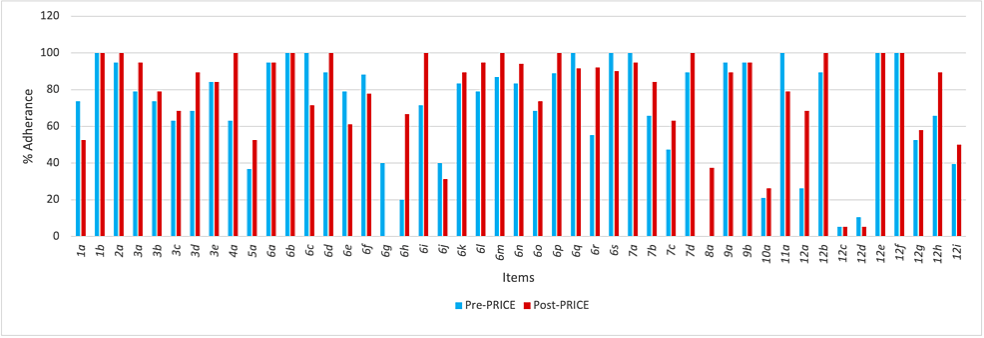
Chart tabulating the percentage adherence of endodontic case reports to PRICE guidelines before and after its release in 2020. PRICE: Preferred Reporting Items for Case reports in Endodontics

Figure 1 depicts the percentage adherence of pre-PRICE and post-PRICE period endodontic case reports. The five items with the greatest percentage increase in guideline adherence are outlined in Table 3; the five items with the greatest percentage decrease in guideline adherence are outlined in Table 4.

**Table 3:** Relative percentage increase in the adherence of case reports to PRICE 2020 reporting item criteria. PRICE: Preferred Reporting Items for Case reports in Endodontics

Item	Description	Relative Percentage Increase in Adherence Pre- Versus Post-PRICE
Item 6h	Case report information: the psychosocial history of the patient if associated with the primary complaint must be provided, if relevant	+233.3
Item 12a	Quality of images: the details of the equipment, software and settings used to acquire the image(s) must be described in the text or legend	+160.0
Item 6r	Case report information: the assessment method(s) used to determine the clinician‐assessed and patient‐assessed treatment outcomes and their results must be provided	+66.7
Item 4a	Introduction: a background summary of the case(s) with relevant information must be provided	+58.3
Item 5a	Informed consent: a clear statement that informed, valid consent was obtained from the patient(s) must be provided	+42.9

**Table 4:** Relative percentage decrease in the adherence of case reports to PRICE 2020 reporting item criteria. PRICE: Preferred Reporting Items for Case reports in Endodontics

Item	Description	Relative Percentage Decrease in Adherence Pre- Versus Post-PRICE
Item 6g	Case report information: the family history of the patient if associated with the primary complaint must be provided, if relevant	-100.0
Item 12d	Quality of images: the resolution and any magnification of the image(s) or any modifications/enhancements (e.g. adjustments for brightness, colour balance, magnification, image smoothing and staining) that were carried out must be described in the text or legend	-50.0
Item 6c	Case report information: the ethnicity of the patient(s) must be provided, if relevant	-28.6
Item 6e	Case report information: the medical history of the patient(s) must be provided, if relevant	-22.6
Item 6j	Case report information: extra‐oral findings must be provided, if relevant	-21.9

## Discussion

Reporting criteria are an important intervention to improve consistency and clarity in the narration of case reports, as has previously been demonstrated with case reporting (CARE [3]) and case series reporting (SCARE [4]) guidelines. This investigation identified a 7.9% overall improvement in adherence to the PRICE guideline following their publication in 2020, elucidating a promising early impact of the guidelines. Although there was an improvement in adherence for the majority of reporting items, few items experienced a fall in compliance (13 items: 1a, 6c, 6e, 6f, 6g, 6j, 6q, 6s, 7a, 9a, 11a, 12c and 12d; Table 1). Most notable of such items is ‘case report information’ (item 6); a thorough, detailed case presentation is an essential component of the case report, and current evidence suggests that the endodontic community is lacking in this domain. Berlin-Broner and Levin reported similar findings in a large five-year evaluation of case reporting adherence to PRICE, identifying lowest scoring items as 6c, 6g, 6h, 6i, 8a, 12c and 12d [8]. Such findings heed the endodontic community not only to read PRICE prior to and during the writing of manuscripts but also to use it as a final screening checklist prior to submission.

PRICE is a substantial and comprehensive guideline with 47 reporting items, far greater than any other case reporting guideline currently available (CARE: 30 individual items [3]; SCARE: 38 individual items [4]). An agreement between the two independent panels of dentists was moderate for both pre-PRICE (ICC: 0.673; p=0.011) and post-PRICE (ICC: 0.742; p=0.003) periods, indicating that there is a small inter-rater variation in the interpretation of the guideline, particularly in elucidating the non-applicability (NA) of reporting items. Further clarity may be required to settle the potential differing interpretations of this aspect of the guideline.

An international, inter-journal, collaborative effort is needed to enhance awareness and adherence to PRICE in order to improve the universal reporting of endodontic case reports. This could be achieved by implementing the guideline as a primary screening tool at journal submission for authors, reviewers and editors. Currently, the International Endodontic Journal is the only endodontic journal that has updated its author guidelines to incorporate PRICE as a submission guide. Greater exposure to PRICE is needed within the endodontic community to establish comprehensive, transparent and educative case reporting practices. Therefore, it is strongly encouraged for authors to read Nagendrababu et al.’s explanation of the PRICE case reporting items to improve writing practice [10]. PRICE checklists and flowcharts can be downloaded by authors from the ‘The Preferred Reporting Items for study Designs in Endodontology’ (PRIDE) website [49]. Further studies are required to assess adherence following a wider acceptance and implementation of the PRICE guideline by endodontic journals internationally.

Limitations

There are some limitations to this study that must be considered. A short sample period was used, comparing the adherence of CRs in the years directly before and after the guideline was released. As such, a significant portion of the endodontic community may still be unaware of PRICE. Furthermore, the panels were not blinded to whether the papers were published prior to or following PRICE. CRs can be defined as articles describing up to four cases [1], but the present study only included CRs describing a maximum of one case to provide greater consistency in adherence grading between panels. There is also some variation in the proportion of journals in which reviewed articles were published in the before-and-after samples; this may affect the validity of the results as journals may have different processing guidelines. There is potential that reporting practices of articles describing multiple cases differ to those only reporting one case; further research is warranted to discern the reporting practices of such CRs and by extension endodontic case series.

## Conclusions

Early indications demonstrate that the PRICE 2020 guideline has resulted in a modest improvement in the reporting of endodontic case reports. Greater exposure and a wider acceptance and implementation of the guideline in endodontic journals are needed to improve adherence to the novel guideline. Further studies are required in the future to assess PRICE 2020 adherence.
